# Delirium and Its Association with Short- and Long-Term Health Outcomes in Medically Admitted Patients: A Prospective Study

**DOI:** 10.3390/jcm12165346

**Published:** 2023-08-17

**Authors:** Aisha Ramadhan Al Huraizi, Juhaina Salim Al-Maqbali, Rajaa Saleh Al Farsi, Khalfan Al Zeedy, Taif Al-Saadi, Noof Al-Hamadani, Abdullah M. Al Alawi

**Affiliations:** 1 Department of Medicine, Sultan Qaboos University Hospital, Muscat 123, Oman; a.alhuraizi@squ.edu.om (A.R.A.H.); khalfanalzidii@gmail.com (K.A.Z.); dr.abdullahalalawi@gmail.com (A.M.A.A.); 2 Department of Pharmacy, Sultan Qaboos University Hospital, Muscat 123, Oman; 3 Department of Pharmacology and Clinical Pharmacy, College of Medicine and Health Science, Sultan Qaboos University, Muscat 123, Oman; 4 Internal Medicine Residency Training Program, Oman Medical Specialty Board, Muscat 130, Oman; rajaa-alfarsi@hotmail.com; 5 College of Medicine, Royal College of Surgeons, D02 YN77 Dublin, Ireland

**Keywords:** delirium, elderly, inpatient, mortality, survival

## Abstract

Background: Delirium is highly prevalent among elderly hospitalized patients in various healthcare settings. This study aimed to assess the impact of delirium on short- and long-term health outcomes. Methods: A prospective cohort included medically ill patients (≥65 years) admitted to a tertiary healthcare facility. Delirium was screened using the 3-Minute Diagnostic confusion assessment method (3D-CAM). Results: During hospitalization, 53.8% (*n* = 153/284) had delirium. Patients with delirium had a longer length of hospital stay (LOS) (7 vs. 5 days; *p* < 0.01) compared to patients without delirium. Delirium caused a higher frequency of high-dependency unit (HDU) or intensive care unit (ICU) admission (*p* < 0.01) and an increased incidence of hospital-acquired complications, including infections (*p* = 0.03), pressure injuries (*p* = 0.01), and upper gastrointestinal bleeding (*p* < 0.01). Inpatient all-cause mortality was higher in patients with delirium than those without delirium (16.3% vs. 1.5%; *p* < 0.01). Patients with delirium had higher rates of 90-day all-cause mortality (25.4% vs. 8.4%; *p* < 0.01) and 1-year all-cause mortality (35.9% vs. 16%; *p* < 0.01) compared to patients without delirium. Patients with delirium exhibited shorter survival periods at 90 days and 1 year compared to patients without delirium with a hazard ratio (HR) = 3.41, 95% CI: 1.75–6.66, *p* < 0.01 and HR = 2.64, 95% CI: 1.59–4.37, *p* < 0.01, respectively. Conclusions: Delirium is associated with serious short-term and long-term clinical consequences. Early recognition, prevention, and targeted interventions addressing reversible risk factors are crucial. Further research is warranted to explore effective strategies for delirium management in general medical wards.

## 1. Introduction

Delirium is highly prevalent among elderly patients admitted to various healthcare settings, with rates reaching up to 82% in mechanically ventilated patients in an ICU setting [1]. It is prevalent in ICU, palliative care, and post-hip-surgery patients [2,3]. A recent prospective study conducted in Oman revealed that delirium affects 55% of elderly patients admitted to medical wards, often going unrecognized by healthcare providers [4].

Delirium is classified into hypoactive and hyperactive subtypes, with some patients having a combination of both, known as mixed delirium. Hyperactive delirium is characterized by increased agitation and restlessness, while hypoactive delirium presents as lethargy and reduced responsiveness [5]. Delirium in the elderly has been consistently linked to poor health outcomes [6]. A meta-analysis revealed that delirium increases the risk of death at 22 months of follow-up, with a hazard ratio (HR) of 1.95 [7]. Unlike many other medical conditions, the mortality associated with delirium has not decreased over the past three decades [8].

Also, delirium is associated with prolonged hospital stays, higher hospital-related complications, increased readmission rates [9], and worse functional and cognitive decline, along with dementia development [10,11]. In addition, delirium consequences affect patients and their families and increase healthcare costs [2]. All of this puts a significant burden on the patients, families, and healthcare system.

Previous studies on delirium have primarily focused on critically ill patients admitted to the ICU [12], with fewer studies examining the health outcomes of delirium in elderly patients admitted to general medical wards [13,14,15]. This study serves as a follow-up to a recently published study of delirium’s prevalence and risk factors among medically hospitalized patients (≥65 years) [4]. We aimed in this study to evaluate the impact of delirium on short- and long-term health outcomes in medically hospitalized elderly patients (≥65 years) in a tertiary healthcare facility. Short-term health outcomes include the length of hospital stay (LOS), the need for high-dependency unit (HDU) or intensive care unit (ICU) admission, the occurrence of hospital-acquired complications, and in-hospital mortality. In comparison, the long-term health outcomes include 90-day all-cause mortality, 90-day readmission, and 1-year all-cause mortality.

## 2. Materials and Methods

### 2.1. Study Population and Design

This prospective cohort study was conducted at Sultan Qaboos university hospital (SQUH) from the 1st of January 2022 to the 31st of May 2023. SQUH, an academic tertiary hospital with 600 beds, is a referral center providing comprehensive care across various medical specialties to patients from all regions of Oman [16]. 

The study included all patients admitted either due to emergency or electively to medical wards who were 65 years of age and above, including those admitted to the medical high-dependency unit. Patients admitted directly to intensive care, hematology, cardiology, and surgical units were not screened. We excluded hematology patients due to the complexity of their hospital stay and the potential for their course of illness to act as a confounder with the associated clinical outcomes. On the other hand, cardiology patients were prone to undergoing surgical procedures during admission, necessitating their exclusion to maintain a focused study population. We excluded patients with language barriers, aphasia, and patients who refused to participate. We also excluded patients admitted with COVID-19 infection.

### 2.2. Assessment Tools and Data Collection

Data were collected prospectively by interviewing patients and their relatives and reviewing their medical records. All patients included in the study were screened for delirium by a research assistant within 24 h of admission, every other day in the first week, and then weekly until delirium occurred or the patient was discharged. Additionally, medical and nursing clinical documentation were reviewed daily, and patients were re-screened if the clinical notes suggested delirium. The 3-Minute Diagnostic confusion assessment method (3D-CAM) was used for screening for delirium. 3D-CAM is a validated bedside assessment tool derived from the extended CAM tool. It is used for screening and diagnoses of delirium with 95% sensitivity and 94% specificity [17,18]. Our study used the Arabic version validated in a similar clinical setting [19]. Patients were screened for pre-existing cognitive impairment and possible dementia using the Informant Questionnaire on Cognitive Decline in the Elderly—Short Form (IQCODE-SF) [20]. The Katz Index of Independence in Basic Activities of Daily Living (BADL) was used to assess the baseline functional status. A score of <6 indicates functional impairment [21]. A previous article by Rajaa et al. provided a comprehensive description of the screening tools utilized in our study [4].

Information about multiple comorbidities (≥3 comorbid conditions), polypharmacy (≥five chronic medications), functional status, pre-existing cognitive dysfunction, and possible dementia was also collected.

For those patients who developed delirium, additional data were collected, including the type of delirium (hyperactive, hypoactive, or mixed), course of recovery (transient, recovered, or persistent), and data on the medications used for the treatment during admission. 

We collected data on inpatient clinical outcomes, including LOS, HDU, or ICU admission requirements, inpatient all-cause mortality, nosocomial infections, venous thromboembolism, pressure injury, and stress upper gastrointestinal bleeding. Patients were then followed post-discharge via phone call, and data related to long-term clinical outcomes were gathered, including 90-day hospital readmission, 90-day all-cause mortality, and 1-year all-cause mortality.

### 2.3. Definitions

- Multiple comorbidities were defined as ≥3 comorbid conditions.
- Polypharmacy was defined as ≥five chronic home medications.
- Pre-existing cognitive dysfunction was defined as patients with a short IQCODE score > 3 [20].
- Hyperactive delirium was defined as patients with agitation or disruptive behavior, hypoactive delirium described patients who appeared apathetic, withdrawn from the environment, or who had depressed levels of arousal, and mixed delirium was defined as patients who demonstrated hyperactive and hypoactive behaviors [22].
- Transient recovery from delirium was defined as delirium that recovered within 24 h, while recovered delirium was defined as delirium recovered via discharge and persistent delirium was defined as delirium present at the time of discharge.
- The educated patient was defined as a patient with a high school education. On the other hand, the uneducated patient was an illiterate person or a patient with an education lower than high school.

### 2.4. Sample Size

The sample size for this study was determined based on the clinical outcome of 1-year all-cause mortality. Previous studies have demonstrated that older patients with delirium have a hazard ratio of 2.64 (95% confidence interval: 1.60–4.35) for 1-year all-cause mortality [23]. We hypothesized that patients with delirium might have an approximately 2.6-fold increase in 1-year all-cause mortality compared to those without delirium during their hospital admission. To achieve a statistical power of 90% and a significance level of 5%, a sample size of 204 patients was initially estimated. However, to account for potential missing data and loss of follow-up, the sample size for this study was increased to 284 patients.

### 2.5. Statistical Analysis

Categorical variables were presented as frequencies and percentages. Based on the Skewness test for normality and Kernel density estimation graph, we determined the normality of our variables. Continuous variables were summarized using either mean and standard deviation (for normally distributed variables) or median and interquartile range (IQR) (for variables with abnormal distribution). The Wilcoxon–Mann–Whitney and Kruskal–Wallis tests were employed to assess differences among continuous groups. For categorical groups, associations were examined using Pearson’s χ^2^ test or Fisher’s exact test in cases with fewer than five observations per cell. Survival analysis assessed 90-day and 1-year all-cause mortality using the Kaplan–Meier method and log-rank tests to compare patients with delirium to those without delirium. A two-tailed significance level was set at *p* < 0.05. STATA version 17.0 (StataCorp, 1985–2021, Stata Statistical Software, College Station, TX, USA) was utilized for all analyses.

### 2.6. Ethical Approval

This study was approved by the Medical Research Ethics Committee of the College of Medicine and Health Sciences of Sultan Qaboos University (REF. NO. SQU-EC/389/2021. MREC #2444). Informed consent was obtained from the patients or their next of kin (if capacity was impaired) before enrollment.

## 3. Results

**Figure 1:**
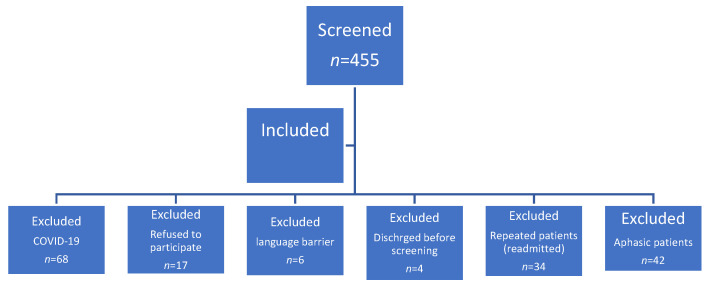
Inclusion and exclusion criteria flow diagram.

A total of 455 patients were initially screened, and after applying the inclusion criteria and excluding those with COVID-19 infection, we identified 284 unique patients (Figure 1). The cohort’s median age was 71 years (IQR: 66–78), with 52.1% (*n* = 148) being female. Among the cohort, 53.8% had delirium, of which 40.1% had delirium upon presentation, while 13.7% developed delirium during hospitalization. The overall mean length of hospital stay was 6 days (IQR: 3–9). Factors s associated with a higher incidence of delirium included a lack of formal education (*p* < 0.01), pre-existing functional impairment (*p* < 0.01), pre-existing cognitive impairment (*p* < 0.01), presence of multiple comorbidities (≥3) (*p* < 0.01), and polypharmacy (≥5) (*p* < 0.01) (Table 1).

Patients with delirium had a longer LOS than patients without delirium (7 (IQR 4–13) vs. 5 (IQR 3–8) days; *p* < 0.01). Also, patients with delirium required ICU (*p* < 0.01) or HDU (*p* < 0.01) admission more frequently than patients without delirium. Additionally, patients with delirium had a higher incidence of nosocomial complications (*p* < 001), pressure injury (*p* = 0.01), hospital-acquired infections (*p* = 0.03), and upper gastrointestinal (GI) stress ulcers (*p* < 0.01). Furthermore, inpatient all-cause mortality was significantly higher in patients with delirium compared to those without delirium (16.3% vs. 1.5%; *p* < 0.01) (Table 1). Patients diagnosed with delirium had higher rates of 90-day all-cause mortality (25.4% vs. 8.4%; *p* < 0.01) and 1-year all-cause mortality (35.9% vs. 16%; *p* < 0.01) compared to those without delirium (Table 1).

The 90-day readmission was studied among the alive patients at 90 days post-discharge (total *n* = 234). However, the difference was not apparent when comparing patients with delirium and without delirium (*n* = 36/114 (31.58%) vs. *n* = 31/120 (25.83%); *p* = 0.33).

Patients with mixed delirium tended to have a longer LOS than patients with hypoactive or hyperactive delirium (*p* = 0.05). However, the difference was not apparent with the other clinical outcomes studied (Table 2).

Out of the patients with delirium (*n* = 153), 53.6% (*n* = 82) had persistent delirium, which was associated with longer LOS (*p* < 0.01) and higher rates of inpatient all-cause mortality (29.27%), 90-day all-cause mortality (*p* < 0.01), and 1-year all-cause mortality (*p* < 0.01) (Table 3).

Most patients with delirium (72.6%) did not receive any specific medications for their condition. Only 27.45% of patients were given medications for delirium, and the class of medications used was as follows: benzodiazepines (78.57%), atypical antipsychotics (26.19%), typical antipsychotics (7.14%), and tricyclic antidepressants (9.52%).

The lack of treatment was not associated with significant differences in inpatient or long-term clinical outcomes (Table 4).

**Figure 2:**
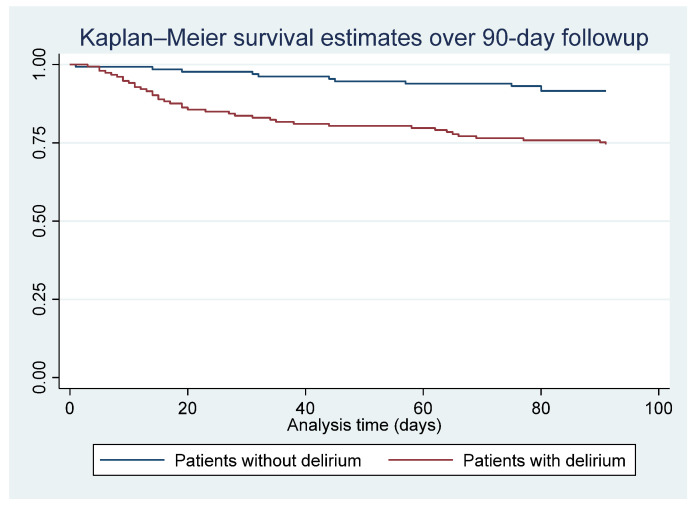
Survival analysis of patients with delirium compared to patients without delirium over 90 days (total mortality: 50). HR: 3.41 (95% CI: 1.75–6.66); *p* < 0.01.

Figure 2 displays the survival analysis, comparing patients with delirium to those without delirium over 90 days. Among the total of 50 deaths during the follow-up, patients with delirium exhibited significantly shorter survival periods compared to patients without delirium (hazard ratio (HR) = 3.41, 95% CI: 1.75–6.66; *p* < 0.01).

**Figure 3:**
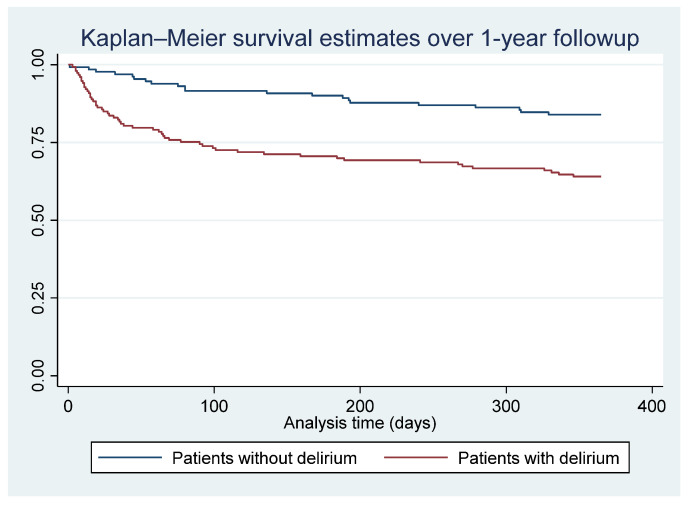
Survival analysis of patients with delirium compared to patients without delirium over 1-year period (total mortality: 76). HR: 2.64 (95% CI: 1.59–4.37); *p* < 0.01.

Similarly, Figure 3 depicts the survival analysis, comparing patients with delirium to those without delirium over 1 year. Among the total of 76 mortalities during the follow-up, patients with delirium had a lower probability of survival than patients without delirium (HR = 2.64, 95% CI: 1.59–4.37; *p* < 0.01).

## 4. Discussion

We believe this study is one of the few studies from the Middle East and North Africa region (MENA) that looks into delirium in elderly patients admitted to medical wards and intensive care units [4,19,24,25]. And, it is the first study to investigate the health outcomes associated with delirium in medically admitted patients in the MENA region. The findings of this study show the substantial link between delirium and poor health outcomes, extending over both the short and long term. The one-year follow-up revealed a high mortality rate among patients with delirium during hospitalization.

In our cohort, 53.8% of elderly patients developed delirium during admission. This is a high rate compared to other studies. The previously reported prevalence of delirium ranged between 11% and 42% in medical wards [9]. The reported incidence in medical wards in two neighboring countries was 21.8 and 17.5% in Saudi Arabia and Iran, respectively [19,25]. We attribute this to several factors, including the complexity of our medical patients, as this tertiary hospital usually admits severely ill patients and those transferred from other institutions. Most of these patients have several risk factors for delirium, like dementia, multiple comorbidities, and polypharmacy, which are known risk factors [4]. Moreover, we included older patients than in other studies, which contributed to the higher rate of delirium (65 years vs. 60 years) [14,19]. In addition, we included high-dependency patients who might have been considered in ICUs in other hospitals. Finally, there is the setting of our hospital, where crowded beds, the noisy environment, and the lack of daylight exposure all contributed to the high incidence of delirium [26]. Although we conducted patient screenings on alternate days, it is important to note that we reviewed both medical and nursing notes daily, and we captured any potential indicators of delirium, such as terms like “sleepy”, “confused”, “aggressive”, “uncooperative”, “agitated”, and others, which prompted screening for delirium. The observed incidence of delirium in our study appears to be relatively high, which might be due to the unique characteristics of our patient cohort, the specific minimum age criteria that we employed for inclusion, and the distinctive attributes of our hospital settings, as well as our deliberate inclusion of high-dependency patients.

In our study, patients with delirium had a prolonged length of hospital stay compared to those without delirium, consistent with the findings from previous studies [27,28]. A large-scale study conducted in the UK found that delirium increased the hospital stay by an average of 3.45 days [29]. The increased length of stay associated with delirium is likely attributed to a higher risk of complications, the need for high acuity care such as ICU or HDU, and the time required for patients to regain the cognitive and physical function necessary for discharge from acute care [28].

Furthermore, delirium is linked to a higher rate of hospital-acquired complications, including infections, bed sores, and incontinence [2,15,30]. It is also associated with worse functional decline upon discharge and a higher rate of transfer to nursing homes [2,30]. Our study aligns with these findings, as patients with delirium showed a higher incidence of hospital-acquired infections, bed sores, and stress ulcers. The longer hospital stay associated with delirium is a strong reason for developing hospital-acquired infections. Moreover, our study’s people with delirium were older, had multiple comorbidities, and were on polypharmacy. This is likely a contributing factor to the development of complications. Additionally, the delirium group had worse functional activity as a baseline, putting them at risk of bed sores, especially if combined with acute illness, further limiting their activity. Moreover, patients with delirium require more care in high-dependency and intensive units, and so they are at greater risk of stress ulcers and overall complications.

Additionally, our study revealed a significant association between the development of delirium and the need for HD or ICU care. This might be linked to the course of complications that patients with delirium experience during admission and the associated short inpatients’ clinical outcomes (nosocomial complication, infections, pressure injuries, and upper GI ulcers). Numerous studies have shown that delirium is common in the ICU [31,32,33]. However, to the best of our knowledge, there are a lack of data exploring delirium as a risk factor for ICU or HD admission. Hence, we recommend further studies to evaluate delirium as a potential independent risk factor for ICU/HD admission. Also, this finding emphasizes the importance of the prevention and early recognition of delirium upon hospitalization.

While many studies have reported a higher readmission rate associated with delirium [7,9], our findings do not demonstrate this relationship. There is an overall elevated hospital readmission rate among both groups with delirium and without delirium, which was previously observed in patients admitted under the care of the General Internal Medicine Unit at SQUH, and this could be attributed to the tertiary hospital setting, where more severely ill patients with complex medical conditions are usually admitted [16,34].

Our study observed a significant mortality rate among patients with delirium, with around 16% dying during the same hospital admission. These findings are consistent with previous studies, which have also reported varying death rates upon discharge ranging from 14.5% to 37% [9]. Previous studies showed that delirium is associated with an increased risk of death independent of age, gender, comorbidities, illness severity, and baseline dementia [7]. The increased mortality rate associated with delirium extended beyond the hospitalization period in our study. Within one year, over one third of patients in the delirium group died during the follow-up period. This finding aligns with previous research that has demonstrated the negative impact of delirium on mortality outcomes [7,35,36]. A meta-analysis revealed that patients with delirium had a higher risk of death, with 37.9% of individuals with delirium dying after an average follow-up of 11 months (OR of 1.71) [7]. 

In this study, persistent delirium at discharge was associated with a higher LOS during admission, and following patients post-discharge and re-scoring them for delirium are essential for this category of patients to allow for controlling the symptoms by adjusting the non-pharmacological and pharmacological therapies, therefore minimizing other long-term clinical outcomes. We also demonstrated that persistent delirium negatively impacted long-term outcomes. A systematic review reported high rates of delirium persistence in elderly patients, with percentages ranging from 21% at six months to 44.7% at one month post-discharge [37]. These findings highlight the prolonged impact of delirium on patients’ health. Persistent delirium has been associated with poor health outcomes, including increased mortality, higher rates of nursing home placement, and a decline in functional and cognitive abilities. The underlying factors contributing to this persistence may include untreated risk factors, medication interactions, and the potential development of dementia [37]. These results highlight the importance of targeted interventions and close monitoring for this specific group of elderly patients to mitigate the long-term consequences of delirium.

Hypoactive delirium is more prevalent and is generally associated with worse outcomes than hyperactive delirium. Studies have reported an association between hypoactive delirium and increased mortality, although findings have been inconsistent in some cases [38,39]. Notably, the mixed subtype of delirium, which combines features of both hypoactive and hyperactive delirium, is often overlooked. The dominance of hypoactive symptoms in this subtype can contribute to delays in diagnosis, while managing hyperactive delirium may necessitate pharmacological intervention. However, this approach carries the risk of oversedation and can lead to a prolonged LOS. 

Delirium is widely believed to have multiple contributing factors, compelling a multidisciplinary approach to its management. This approach requires early recognition, addressing underlying risk factors, and implementing targeted pharmacological and non-pharmacological interventions. The primary focus of managing delirium’s symptoms lies in non-pharmacological and supportive methods. However, in cases where symptoms are distressing or pose risks to the patient and others, pharmacological management may be necessary when non-pharmacological interventions prove to be ineffective [40]. 

In our study, using pharmacological management to control delirium did not affect any measured outcomes. Although various medication groups are widely used, no single option has proven its ability to prevent or cure delirium during hospitalization, and their effects are inconsistent [32,41,42]. In our cohort, the use of benzodiazepine accounted for 79% of all of the medications used to treat delirium, which is no longer recommended in the treatment of delirium because of the oversedation effect and its association with worsening cognition [41,43]. These findings reflect the need for increasing clinicians’ awareness about delirium management and the best pharmacological options. Typical antipsychotics (namely haloperidol) and atypical antipsychotics (namely quetiapine) are commonly used medications with acceptable safety profiles when used in low doses [43]. Alpha-2 receptor agonists (namely dexmedetomidine) were found to be preventive and a more potent option for treating delirium in ICU patients [43]. 

Preventive measures play a crucial role and form the cornerstone for the management of delirium by targeting reversible risk factors, including environmental interventions such as quiet time, sleep enhancement, family support, effective communication, and appropriate management of pain and dyspnea [33]. Other emerging areas for the prevention of hospital-induced delirium and particularly in ICU settings include the short-term use of melatonin or aripiprazole, which are showing a promising reduction in the incidence of delirium. However, their role is inconclusive and lacks confirmatory evidence [41,42].

This study has many strengths as it provides a comprehensive understanding of the high prevalence of delirium and its impact on health outcomes in patients hospitalized in medical wards. The study employed validated assessment tools, had a relatively large sample size, and included a long-term follow-up period of one year. Multiple outcomes were assessed, including LOS, ICU, or HDU admission, in-hospital complications, and mortality. These strengths enhance the significance and reliability of the findings, highlighting the need for preventive measures and targeted interventions to mitigate the adverse effects of delirium in this population. Further studies are necessary to strengthen our findings and hence to develop new approaches for prevention and treatment. We suggest conducting randomized controlled trials to investigate potential strategies for preventing or treating delirium. Moreover, we recommend emphasizing non-pharmacological methods in our clinical practices and establishing a dedicated geriatric ward and team specifically caring for elderly patients.

We identified a few limitations to the study. First, being a single-center study and given that the life expectancy in the Omani population is lower (75 years old) compared to other developed countries, our cohort median age was 71, which might influence the study’s generalizability. We only found a single incidence of falls, which was not representative, making the comparison among the delirium groups challenging. We only identified 11 patients with hyperactive delirium; this small number might have affected the study’s ability to find significant differences. We did not assess post-discharge decline in functional impairment nor the incidence of persistent delirium, which might be associated with worsening clinical outcomes. The sample size was not calculated for the readmission rate, which might have contributed to the small difference in the 90-day readmission rate among the alive patients with and without delirium.

## 5. Conclusions

Delirium has serious short-term and long-term negative clinical consequences, such as prolonged LOS, higher inpatient complications, higher all-cause mortality rates, and shorter survival probability. Early recognition, prevention, targeted interventions addressing reversible risk factors, and emphasizing non-pharmacological methods are crucial. Further research is necessary to gain a comprehensive understanding of delirium’s effects on health outcomes and to develop new approaches for prevention and treatment. 

## Figures and Tables

**Table 1:** Characteristics, inpatient, and long-term clinical outcomes of the patients stratified according to the incidence of delirium (*n* = 284).

	Total 284 (100%)	No Delirium 131 (46.13%)	Delirium 153 (53.87%)	*p*-Value
Age (IQR), years	71 (66–78)	68 (64–74)	75 (68–80)	<0.01
Female	148 (52.11%)	59 (45.04%)	89 (58.17%)	0.027
Not educated	219 (77.11%)	88 (67.18%)	131 (85.62%)	<0.01
Functional impairment (BADL < 6)	191 (67.25%)	57 (43.51%)	134 (87.58%)	<0.01
Pre-existing cognitive dysfunction	63 (22.18%)	10 (7.63%)	53 (34.64%)	<0.01
Multiple comorbidities (≥3)	239 (84.15%)	100 (76.34%)	139 (90.85%)	<0.01
Polypharmacy (≥5)	260 (91.55%)	112 (85.50%)	149 (96.73%)	<0.01
**Inpatient clinical outcomes**				
LOS (IQR), days	6 (3–9)	5 (3–8)	7 (4–13)	<0.01
ICU admission	17 (5.99%)	2 (1.53%)	15 (9.80%)	<0.01
HD admission	35 (12.32%)	4 (3.05%)	31 (20.26%)	<0.01
Nosocomial complication	53 (18.79%)	11 (8.53%)	42 (27.45%)	<0.01
Pressure injury	15 (5.35%)	1 (0.78%)	14 (9.15%)	<0.01
Hospital-acquired infection	38 (13.48%)	11 (8.53%)	27 (17.65%)	0.03
Stress upper GI ulcers	13 (4.61%)	0	13 (8.50%)	<0.01
Venous thromboembolism	4 (1.42%)	1 (0.78%)	3 (1.96%)	0.628
**All-cause mortality**				
Inpatient all-cause mortality	27 (9.51%)	2 (1.53%)	25 (16.34%)	<0.01
90-day all-cause mortality	50 (17.61%)	11 (8.40%)	39 (25.49%)	<0.01
1-year all-cause mortality	76 (26.76%)	21 (16.03%)	55 (35.95%)	<0.01

IQR: Interquartile Range; LOS: Length of hospital stay; ICU: Intensive care Unit; HDU: High dependency Unit; GI: gastrointestinal; BADL: Basic Activities of Daily Living.

**Table 2:** Clinical outcomes associated with delirium were stratified according to type of delirium (*n* = 153).

Clinical Outcomes *n* (%) unless Specified Otherwise	Total 153 (100%)	Hypoactive Delirium 102 (66.67%)	Hyperactive Delirium 11 (7.19%)	Mixed Delirium 40 (26.14%)	*p*-Value
LOS (IQR), days	7 (4–13)	7 (4–12)	6 (4–6)	9 (6–16)	0.05
Inpatient all-cause mortality	25 (16.34%)	19 (18.63%)	1 (9.09%)	5 (12.50%)	0.62
90-day all-cause mortality	39 (25.49%)	28 (27.45%)	1 (9.09%)	10 (25.00%)	0.47
1-year all-cause mortality	55 (35.95%)	40 (39.22%)	2 (18.18%)	13 (32.50%)	0.38

**Table 3:** Clinical outcomes associated with delirium were stratified according to the course of delirium (*n* = 153).

Clinical Outcomes *n* (%) unless Specified Otherwise	Total 153 (100%)	Persistent Delirium 82 (53.59%)	Transit Delirium 29 (18.95%)	Recovered Delirium 42 (27.45%)	*p*-Value
LOS (IQR), days	7 (4–13)	9 (5–16)	5 (2–6)	7 (4–9)	<0.01
Inpatient all-cause mortality	25 (16.34%)	24 (29.27%)	1 (3.45%)	0	<0.01
90-day all-cause mortality	39 (25.49%)	33 (40.24%)	2 (6.90%)	4 (9.52%)	<0.01
1-year all-cause mortality	55 (35.95%)	44 (53.66%)	4 (13.79%)	7 (16.67%)	<0.01

**Table 4:** Clinical outcomes associated with delirium were stratified according to the treatment (*n* = 153).

Clinical Outcomes *n* (%) unless Specified Otherwise	Total 153 (100%)	Not-Administered Delirium Medications 111 (72.55%)	Administered Delirium Medications 42 (27.45%)	*p*-Value
LOS (IQR), days	7 (4–13)	7 (4–13)	8 (5–13)	0.57
Inpatient all-cause mortality	25 (16.34%)	17 (15.35%)	8 (19.05%)	0.58
90-day all-cause mortality	39 (25.49%)	30 (27.03%)	9 (21.43%)	0.48
1-year all-cause mortality	55 (35.95%)	41 (36.94%)	14 (33.33%)	0.68

## Data Availability

Data is available from the corresponding author on request.
